# Influence of *Lactobacillus kefiri* on Intestinal Microbiota and Fecal IgA Content of Healthy Dogs

**DOI:** 10.3389/fvets.2020.00146

**Published:** 2020-04-02

**Authors:** Alba Gaspardo, Augusta Zannoni, Silvia Turroni, Monica Barone, Maria Chiara Sabetti, Renato Giulio Zanoni, Monica Forni, Patrizia Brigidi, Marco Pietra

**Affiliations:** ^1^Department of Veterinary Medical Sciences, University of Bologna, Ozzano Dell'Emilia, Italy; ^2^Health Sciences and Technologies - Interdepartmental Center for Industrial Research (CIRI-SDV), University of Bologna, Bologna, Italy; ^3^Unit of Microbial Ecology of Health, Department of Pharmacy and Biotechnology, University of Bologna, Bologna, Italy; ^4^Interdepartmental Centre for Agri-Food Industrial Research, University of Bologna, Bologna, Italy

**Keywords:** dog, stool, gut microbiota, probiotic, IgA

## Abstract

The increasing incidence of gastrointestinal tract pathologies in dogs and the worrisome topic of antibiotic resistance have raised the need to look for new therapeutic frontiers. Of these, the use of probiotics represents a potential therapeutic alternative. *Lactobacillus kefiri* (*Lk*) is a species of *Lactobacillus* isolated from kefir. Previous studies have demonstrated that its administration in mice downregulates the expression of proinflammatory mediators and increases anti-inflammatory molecules in the gut immune system. It also regulates intestinal homeostasis, incrementing immunoglobulin A (IgA) secretion. Since *Lk* has never been studied as a single probiotic in dogs, the aim of this study was to evaluate the safety of *Lk* in dogs, and its effect on IgA secretion and on intestinal microbiota composition. Ten healthy dogs without a history of gastrointestinal diseases were included. The dogs received *Lk* at a dose of 10^7^ live microorganisms orally, once daily for 30 days. The fecal samples were tested before administration, in the middle, at the end, and 30 days after discontinuation. The IgA secretion concentration and the microbiota composition were evaluated on the fecal samples. The results in this study suggested that *Lk* did not influence the concentration of IgA, nor significant changes of the intestinal microbiota were observed during and after the treatment. Therefore, additional studies are needed to investigate if a higher daily dosage of *Lk* can influence the intestinal homeostasis of dogs.

## Introduction

In recent years, intestinal microbiota has become increasingly relevant for veterinary scientists and has been studied for its role on the welfare of the host (1). It is an ecosystem including mainly bacteria, but also archaea, fungi, protozoa, and viruses, which plays several roles in the host physiology by means of a range of metabolic and immunological interactions. In fact, this complex ecosystem helps in the digestion of food by assisting the absorption and metabolism of nutrients, and has trophic and protective functions (2). It defends the gastrointestinal tract (GIT) against pathogenic organisms, promotes mucus production and enterocyte turnover, and modulates host immune development and functionality (3). In particular, commensal bacteria provide intestinal immune protection by regulating, among other things, the secretion of IgA, the lack of which seems to be correlated with chronic enteropathies in dogs (4, 5).

Canine chronic enteropathies, categorized into four classes (food responsive; antibiotic responsive; immunosuppressant responsive; nonresponsive enteropathy) according to the response to treatment, are multifactorial diseases where host genetic factors, the immune system, and indigenous intestinal bacteria are supposed to be engaged in intricate interactions (6, 7).

Dogs affected by antibiotic responsive enteropathy (ARE) are generally young, predominantly belong to large breeds, and show remission of clinical signs following antimicrobial administration (metronidazole, tylosin, doxycycline, rifaximine) (8, 9). It is thought that antimicrobials are able to modify the intestinal microbial population by counteracting its imbalances (i.e., dysbiosis); however, although a short-term response to metronidazole and tylosin has been reported, very few papers have described the long-term control of ARE (9–11).

Moreover, there is now evidence that prolonged treatment with antibiotics, particularly with metronidazole, can lead to permanent unfavorable changes in the microbiota, promoting antimicrobial resistance, currently one of the most important threats to public health (12, 13).

Therefore, the increasing incidence of GIT pathologies and the worrisome topic of antibiotic resistance create the need for new therapeutic options (14), and toward replacing antibiotics with (a) probiotics (live microorganisms that confer a healthy benefit on the host); (b) prebiotics (a substrate selectively utilized by host microorganisms useful for reestablishing a eubiotic microbiota layout); (c) synbiotics (a mixture of probiotics and prebiotics having a synergistic action on host health); and (d) postbiotics (soluble factors or metabolic byproducts, secreted by live bacteria or released after bacterial lysis expressing biological activity in the host).

Lactic acid bacteria (LAB), including *Lactococcus, Streptococcus, Enterococcus, Pediococcus, Leuconostoc*, and *Lactobacillus*, are probiotic bacteria that are normally part of the intestinal microbiota of dogs and cats (2). *Lactobacillus* species are distributed throughout the canine intestinal tract in varying amounts (2). Several strains of Lactobacilli have specifically been studied for their ability to reduce the number of pathogenic bacteria in the canine intestine (15–20).

Among different naturally fermented foods and their potential probiotic properties, particular attention has recently been focused on kefir, a dairy product that could modulate canine intestinal microbiota if given regularly (21). Kefir has a complex composition of microbial organisms, which includes several species of LAB as well as acetic acid bacteria and yeast (22). *Lactobacillus kefiri* (*Lk*) is a *Lactobacillus* species that has been isolated from kefir (23). Previous studies have demonstrated that its administration in mice downregulates the expression of proinflammatory mediators and increases anti-inflammatory molecules in the gut immune system (24, 25). It also regulates intestinal homeostasis by increasing IgA secretion and mucin production (25). Probiotic properties of *Lk* have also recently been demonstrated in humans (26); however, to the best of the authors' knowledge, the use of *Lk* has never been evaluated in dogs.

The aim of this study was to evaluate the safety and ease of administration of *Lk* in healthy dogs and its ability to impact the intestinal microbiota composition and IgA secretion.

## Materials and Methods

### *Lactobacillus kefiri* Administration

This study is based on the use of a commercial food supplement (Kefibios^®^), provided by the company Hulka S.r.L. (Rovigo, Italy), containing live lactic ferments of *Lk* (LKF01–DSM 32079), currently used as probiotics in human medicine. Copyright permission to publish the product name (Kefibios^®^) was given by the Chief Executive Officer of Hulka S.r.L. The product is marketed in capsules. Following the label of the product, five drops of the solution reconstituted with 6 ml of vegetable oil in prefilled bottles contain ≥10^9^ active fluorescent units (AFUs) of live and viable *Lk* (ISO 19344:2015). The dose corresponds to the human one indicated by the company, regardless of age and body weight (BW). The study, involving 10 healthy privately owned dogs, was conducted at the Veterinary Teaching Hospital (VTH) of our department. The owners were carefully instructed as to how to use and mix the product, and then shake the bottle before each administration. The product was then stored at room temperature between 10°C and 25°C and away from direct light.

The recruitment of the dogs in the study was voluntary and at no cost to the owners. Written informed consent before enrollment in the study was obtained from the owners.

The trial was authorized by the Animal Welfare Committee of the University of Bologna (Protocol No. 3885 of 21/07/2017).

### Animals and Experimental Design

Privately owned dogs of various breeds, genders, and weight, over 1 year of age, were enrolled in the trial. Dogs with any disease in the previous 2 months before the start of the trial were excluded. The inclusion criterion was the absence of antimicrobial or immunosuppressive treatment up to 60 days prior to the start of and during the trial. For inclusion, each dog was evaluated with a clinical examination and a laboratory panel, which included a complete blood count, a serum biochemistry profile, and coprological examination for gastrointestinal parasites.

The body condition score (BCS) was calculated using the 1–9 score proposed by Royal Canine SAS, and the fecal score was evaluated according to the Fecal Score System (FSS 1-7) proposed by Nestlé-Purina Petcare. The BCS was determined as follows: 1–3 = too thin, 4–5 = ideal, and 6–9 = too heavy. The fecal samples were scored as follows: 1 = very hard and dry, leaves no residue on the ground when picked up; 2 = firm, but not hard, pliable, little or no residue on the ground when picked up; 3 = log-shaped, moist surface, leaves residue on the ground, but holds form when picked up; 4 = very moist and soggy, leaves residue on the ground and loses form when picked up; 5 = very moist but has a distinct shape, leaves residue on the ground and loses form when picked up; 6 = has texture, but no defined shape, leaves residue on the ground when picked up; and 7 = watery, no texture, present in flat patches.

Not being experimental animals but privately owned dogs, diet was not standardized during the trial but the previous diet (Table 1) was maintained, and water was supplied *ad libitum*.

**Table 1:** Dogs included in the study.

**Dogs**	**Breed**	**Gender**	**Age**	**Weight**	**BCS**	**Fecal score**	**Diet**
1	Mix breed	C	4y1m	33 kg	6	2	Purina tonus dog chow chicken
2	Mix breed	S	4y0m	18.3 kg	6	2	Purina tonus dog chow chicken
3	Border Collie	F	2y6m	19 kg	4	2	Prolife adult medium chicken and rice
4	Australian shepherd	S	6y0m	23 kg	5	2	Royal canin veterinary diet neutered adult medium dog
5	Border Collie	C	2y6m	20.5 kg	5	2	Farmina ancestral low grain lamb and blueberry
6	Dachshund	F	3y4m	5.6 kg	6	2	Royal canin small dog chicken and rice
7	Border Collie	S	9y7m	19 kg	5	2	Prolife adult medium chicken and rice
8	Labrador retriever	M	2y5m	32.1 kg	5	3	Monge natural superpremium rabbit, rice, and potatoes
9	Mix breed	S	9y8m	7.5 kg	5	3	Royal canin small dog chicken and rice
10	Mix breed	C	5y11m	5.7 kg	5	2	Royal canin small dog chicken and rice

*M, male; C, neutered male; F, female; S, neutered female; BCS, body condition score*.

During the entire experimental time, the dogs received five drops (≥10^9^ AFUs) of *Lk* once daily for 30 days, administered directly in the mouth during the dinner meal.

Clinical examination, BCS, and FSS were performed at inclusion, and, during the trial, respectively, 15, 30, and 60 days after the start of *Lk* administration_._

Samples of fresh feces were collected from each dog on three consecutive days: (a) at T0, before the start of *Lk* administration (days −3, −2, −1); (b) at T15, in the intermediate time of *Lk* administration (days 13, 14, 15); (c) at T30, at the end of *Lk* administration (days 28, 29, 30); (d) at T60, 1 month after the last *Lk* administration (days 58, 59, 60).

The samples were collected by the owner immediately after defecation, immediately stored at −20°C in the owner's household freezer until delivered frozen to the department where they were stored at −80°C until use.

All the samples were analyzed for IgA detection, while, for the gut microbiota, a pool of three samples from each dog at T0, T30, and T60 was prepared and analyzed.

### Fecal Microbiota Analysis

Microbial DNA was extracted from about 250 mg of pooled fecal sample, derived from the experimental points (T0; T30; T60) for each dog, using the repeated bead-beating plus column method, as previously described (27). Briefly, samples were suspended in 1 ml of lysis buffer (500 mM NaCl, 50 mM Tris-HCl, pH 8, 50 mM EDTA, and 4% SDS) and bead-beaten three times in a FastPrep instrument (MP Biomedicals, Irvine, CA, USA) at 5.5 movements/s for 1 min, in the presence of four 3-mm glass beads and 0.5 g of 0.1-mm zirconia beads (BioSpec Products, Bartlesville, OK, USA). After incubation at 95°C for 15 min, samples were centrifuged at 13,000 rpm for 5 min. Two hundred and sixty microliters of 10 M ammonium acetate was added to the supernatant, followed by 5-min incubation on ice and 10-min centrifugation at 13,000 rpm. The supernatant was added with one volume of isopropanol, followed by incubation on ice for 30 min. Precipitated nucleic acids were washed with 70% ethanol, resuspended in 100 μl of TE buffer, and treated with 2 μl of 10 mg/ml DNase-free RNase at 37°C for 15 min. DNA was further purified using the QIAamp Mini Spin columns (QIAGEN, Hilden, Germany) following the manufacturer's instructions. DNA concentration and quality were evaluated using the NanoDrop ND-1000 spectrophotometer (NanoDrop Technologies, Wilmington, DE, USA).

The V3–V4 hypervariable region of the 16S rRNA gene was amplified by using the 341F and 785R primers with Illumina adapter overhang sequences, as previously described (28). PCRs were performed in a final volume of 25 μl, containing 12.5 ng of genomic DNA, 200 nM of each primer, and 2X KAPA HiFi HotStart ReadyMix (Kapa Biosystems, Wilmington, MA, USA), in a Thermal Cycler T (Biometra GmbH, Göttingen, DE) with the following thermal cycle: initial denaturation at 95°C for 3 min, 25 cycles of denaturation at 95°C for 30 s, annealing at 55°C for 30 s and extension at 72°C for 30 s, and a final extension step at 72°C for 5 min. Amplicons were purified using a magnetic bead-based system (Agencourt AMPure XP; Beckman Coulter, Brea, CA, USA). Indexed libraries were prepared by limited-cycle PCR using Nextera technology and further purified as described above. Final libraries were pooled at equimolar concentration, denatured with 0.2 N NaOH, and diluted to 6 pM before loading onto the MiSeq flow cell. Sequencing was performed on Illumina MiSeq platform with a 2 × 250 bp paired-end protocol, according to the manufacturer's instructions (Illumina, San Diego, CA, USA).

Sequencing Reads Were Deposited in the National Center for Biotechnology Information Sequence Read Archive (NCBI SRA; BioProject ID PRJNA 592436).

### Quantification of IgA Fecal Content

Stool samples were thawed and subsequently freeze-dried (Modulyo EF4, 1044, Edwards, Apeldoorn, The Netherlands) for 16 h in order to eliminate the water contained and standardize the subsequent analysis, as reported by Grellet et al. (29). The lyophilized fecal samples were resuspended in 1X PBS (phosphate buffer saline) containing 0.5% Tween20 (Sigma-Aldrich, St. Louis, MO, USA), according to a weight/volume ratio of 100 mg/ml (1:10 dilution) by vortex (3') and then centrifuged at 1,500 × *g* for 10 min. After removing the supernatant, a further centrifugation was carried out at 10,000 × *g* for 20 min, then the aqueous phase was taken and frozen at −20°C until the processing moment. The determination of the IgA secretion amount was carried out by using a commercial kit (dog IgA ELISA Quantitation Set, Bethyl Laboratories Inc., TX, USA; Assay Range: 15.6–1,000 ng/ml), following the manufacturer's protocol.

### Kefibios^®^ Quality Control

In order to confirm the content of *Lk* declared by the manufacturer, we performed an independent assessment of *Lk* concentration in Kefibios^®^ capsules (Bacteriological Laboratory, UNI EN ISO 9001:2015 registration number IT-15164).

Five different batches of the product, acquired in several drugstores, with expiration date from 1 to 2 years with respect to the time of the analysis, were analyzed, and enumeration of viable bacteria was conducted by the plate count method.

Briefly, each sample was firstly solubilized in MRS Broth (Oxoid CM359, Thermo Scientific, MA, USA) and gently shaken at room temperature for 15 min by using an orbital shaker, then serially diluted in the same broth and inoculated onto MRS Agar (Oxoid CM361, Thermo Scientific, MA, USA) plates. Plating was performed in triplicate.

Plates were incubated at 37°C for 72 h under anaerobic atmosphere, and the number of colony-forming units (CFUs) was determined.

The colonies obtained in the tests were identified by the API 50 CHL (BioMerieux, FI, Italy) test, and results are presented as the number of viable cells per capsule and per dose (five drops).

### Bioinformatics and Statistics

For the analysis of fecal microbiota composition and diversity, raw sequences were processed using a pipeline combining PANDAseq (30) and QIIME 1 (31). High-quality reads were binned into operational taxonomic units (OTUs) through an open-reference strategy at 0.97 similarity threshold by using UCLUST (32). Taxonomy was assigned using the RDP classifier and Greengenes as a reference database (release May 2013). All singleton OTUs and chimeras, identified by ChimeraSlayer (33), were discarded. Alpha rarefaction was performed using observed OTUs, Shannon, and the phylogenetic diversity (PD) whole tree metrics, while beta diversity was estimated by computing weighted and unweighted UniFrac distances. For the identification of *Lk*, OTUs assigned to the genus *Lactobacillus* were subjected to BLAST analysis (34).

All statistical analyses were performed in R (version 3.1.3) using the packages vegan, made4, and GraphPad Prism V.5.01 (GraphPad Software, La Jolla, CA, USA).

Assessment of data for normality was carried out by applying the D'Agostino and Pearson Omnibus normality test.

UniFrac distances were used for principal coordinates analysis (PCoA), and the significance of data separation was tested using a permutation test with pseudo-F ratios (function Adonis of vegan). Wilcoxon test for paired data was used to assess significant differences in alpha diversity and taxon relative abundance between groups.

A repeated-measures ANOVA (with Tukey *post hoc* test) was applied to evaluate the differences in BW and fecal IgA content between experimental points (T0, T15, T30, and T60). Friedman test (with Dunn's as *post hoc* test) was applied to evaluate the differences in BCS and FSS between experimental points (T0, T15, T30, and T60).

*p* < 0.05 was considered statistically significant.

## Results

### Animals

Ten healthy adult dogs were included. Of those, one was male, three neutered males, two females, and four neutered females. Mean age was 4.9 ± 2.8 years (range 2–9). Breeds included were mixed breed dogs (*n* = 4), Border Collies (*n* = 3), Labrador Retriever (*n* = 1), Australian Shepherd (*n* = 1), and Dachshund (*n* = 1). Their BW ranged from 5 to 33 kg (18.3 ± 9.84 kg), while their average BCS was normal (range 4–6). Initial FSS was normal in all dogs (range 2–3; Table 1). All dogs had normal hematological and biochemical parameters, and the coprological examination was negative for parasites.

The liquid product containing *Lk* was spontaneously accepted by all the subjects. No clinical signs during the trial (with the exception of dog #6), and up to 1 month later, were reported by the owners.

There were no significant changes in BW and BCS during the study period, nor did the FSS of each animal changed.

Dog #6 developed a urinary tract infection at day 50 of the trial that required antibiotic treatment. This dog's last fecal sample was therefore excluded from the analysis.

### Microbiota Analysis

A total of 1,536,903 high-quality reads (mean ± SD, 52,997 ± 16,302) were obtained and clustered into 2,716 OTUs at 97% similarity.

**Figure 1:**
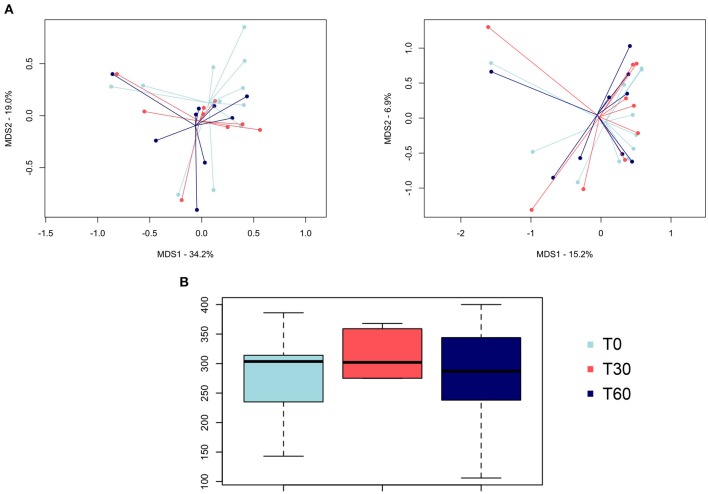
Gut microbiota diversity of healthy dogs following *Lk* administration. **(A)** Principal coordinates analysis of intersample diversity, based on weighted (left) and unweighted (right) UniFrac distances. **(B)** Alpha diversity computed with observed OTU metrics. T0, baseline; T30, after 30 days of *Lk* administration; T60, 1 month after the end of the treatment.

The PCoA of intersample diversity based on weighted and unweighted UniFrac distances showed no significant separation among the study groups (i.e., baseline, end of treatment, and follow-up; *P* = 0.7, permutation test with pseudo-F ratios; Figure 1A). Similarly, no significant differences were found in alpha diversity after *Lk* administration or in the follow-up compared to the baseline (*P* > 0.05, Wilcoxon test; Figure 1B).

**Figure 2:**
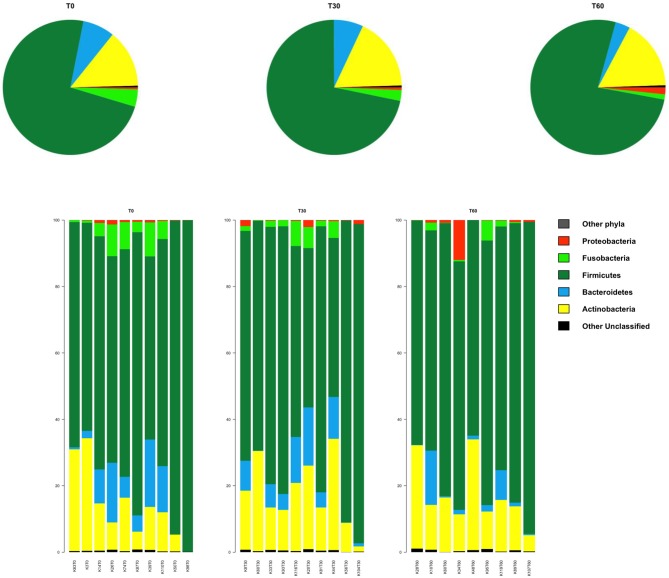
Impact of *Lk* administration on the phylum-level compositional structure of the gut microbiota of healthy dogs. **Top**: pie charts of mean values of relative abundance; **Bottom**: bar plots of individual profiles. T0, baseline; T30, after 30 days of *Lk* administration; T60, 1 month after the end of the treatment.

**Figure 3:**
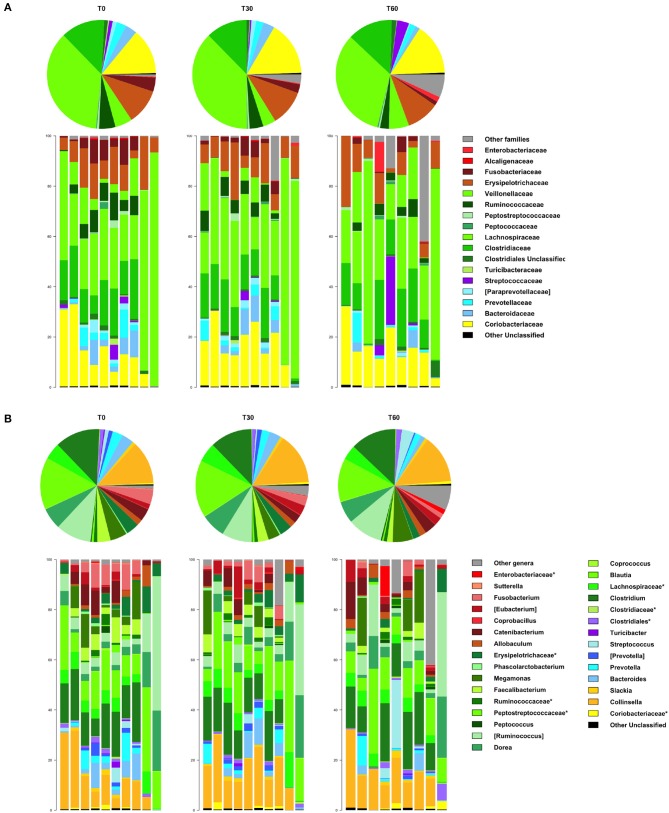
Impact of *Lk* administration on the family- and genus-level compositional structure of the gut microbiota of healthy dogs. Relative abundance profiles at family **(A)** and genus **(B)** level. Top: pie charts of mean values; bottom: bar plots of individual profiles. T0, baseline; T30, after 30 days of *Lk* administration; T60, 1 month after the end of the treatment.

In line with the available literature on the gut microbiota of healthy dogs (35, 36), the phylum-level microbial profiles at the baseline were dominated by Firmicutes (relative abundance, mean ± SEM, 73.1 ± 4.2%), with Actinobacteria (14.2 ± 3.1%), Bacteroidetes (7.8 ± 2.2%), and Fusobacteria (3.9 ± 1.2%) as minor components (Figure 2). *Lachnospiraceae, Coriobacteriaceae, Clostridiaceae*, and *Erysipelotrichaceae* were the major families of the baseline microbiota (relative abundance ≥ 10%; Figure 3A). Consistently, the most represented genera were *Blautia, Clostridium*, and *Collinsella* (Figure 3B). Following *Lk* administration, no significant differences in taxon relative abundance at these phylogenetic levels (i.e., phylum, family, and genus) were observed (Figures 2, 3). One month after the end of the treatment, these changes were no longer detectable.

However, at T60, a decreasing trend in the relative abundances of *Fusobacteriaceae* and *Ruminococcaceae* was found (T0 vs. T60, *P* = 0.11 and 0.15, respectively; Figure 3A). With specific regard to *Lk*, OTUs assigned to this species were not present at T0 but accounted for 10.3% of *Lactobacillus* diversity (and 0.07% of the intestinal ecosystem) after 30 days of *Lk* administration, which then disappear again in the follow-up.

### IgA Content in Fecal Samples

A total of 117 fecal samples were taken and analyzed. Three samples (T58, T59, and T60 from dog #6) were missing because dog #6 was excluded from the trial.

The water content of feces was very similar among all the samples with percentage values of 38.08 ± 4.97 (mean ± SD).

**Figure 4:**
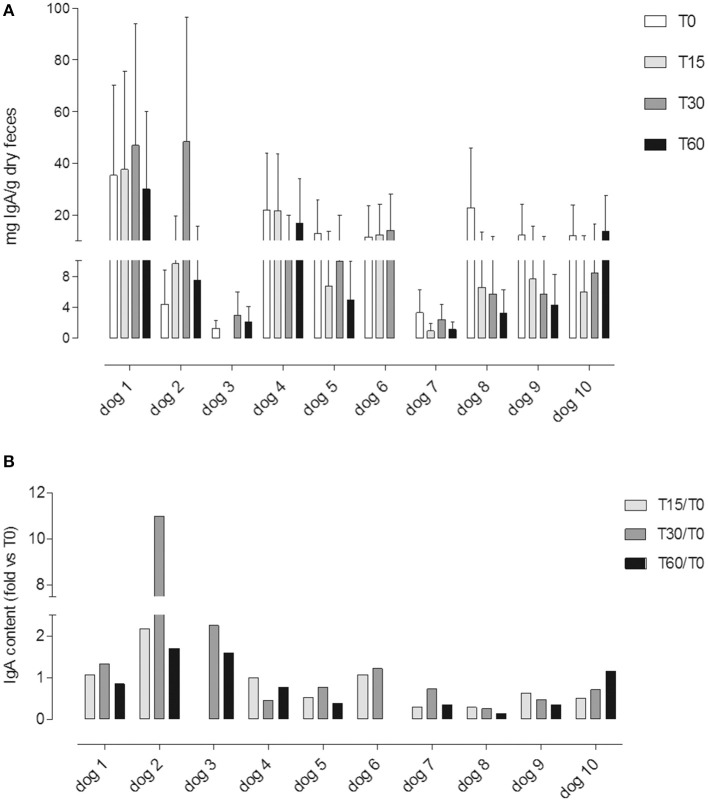
**(A)** Fecal IgA content at different time points (T0; T15; T30; T60); each value (mean ± SD) represents the average of the measurements of the three samples collected in three consecutive days for each experimental point: T0, before the administration, T15 and T30, after 15 and 30 days of *Lk* administration; and T60, 1 month after the last *Lk* administration. The T60 fecal samples of dog #6 were excluded from the analysis due to antibiotic therapy. **(B)** IgA fecal content evaluated by the ratio between the quantity at T15, T30, and T60 and the basal (T0) for each dog.

IgA was detected in all samples except for sample T15 from dog #3 (undetectable <15.6 ng/ml). All dogs have shown a huge intra-individual variability of fecal IgA content among the three following day samples at T0, T15, T30, and T60 (Figure 4A).

Only in four dogs it was possible to observe an increasing trend in the IgA fecal content (dogs 1, 2, 3, and 6; Figure 4A).

In Figure 4B, we showed a ratio between the quantity of IgA fecal content at T15, T30, and T60 and the basal (T0) for each dog.

**Figure 5:**
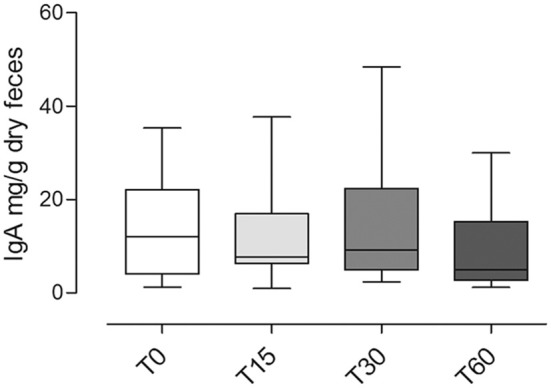
Fecal IgA content at different time points (T0; T15; T30; T60) in dogs included in the trial (mean ± SD). No significant statistical differences were observed (*p* = 0.1).

Overall, the fecal IgA content between different time points did not show any significant differences (*p* = 0.1; Figure 5).

### Kefibios^®^ Quality Control

The number of viable *Lk* per capsule from different product batches varied from 7.73 × 10^7^ CFU to 152 × 10^7^ CFU (81 ± 61 × 10^7^ CFU, mean and standard deviation).

The number of viable *Lk* in the liquid formulation per dose of five drops varied from 0.39 × 10^7^ CFU to 7.55 × 10^7^ CFU (3.2 ± 2.4 × 10^7^ CFU, mean and standard deviation) and was hence 2-log fold lower than the declared concentration (≥10^9^ CFU) by the company.

## Discussion

In recent years, interest in characterizing the canine intestinal microbiota has soared, and therapeutical interventions that can positively influence the microbiota composition and function, specifically identification of novel probiotics, are sought (2, 3, 37).

Several potential probiotics have already been tested in dogs, including bacterial species belonging to the genera *Enterococcus, Lactobacillus, Bifidobacterium*, and *Saccharomyces*, demonstrating their role in the treatment of acute and chronic enteropathies (15–18, 38–40).

Among *Lactobacillus* strain, several species have been already studied (*Lactobacillus acidophilus* DSM13241; *Lactobacillus fermentum* CCM7421; *Lactobacillus animalis* LA4). However, the achieved results are difficult to compare as different dosages (10^7^ CFU/daily dose; 10^9^ CFU/daily dose; 3 to 3.6 × 10^9^ CFU/daily dose) and different forms of application were used, and the duration of administration was also variable (15, 17, 18).

To date, no studies have evaluated the influence of *Lk* on the parameters of intestinal health in dogs.

Only one previous study has assessed the effect of kefir (a fermented dairy product containing *Lk*) on dogs (21). This study demonstrated a significant increase in the fecal LAB:*Enterobacteriaceae* ratio and a decrease in the fecal Firmicutes:Bacteroidetes ratio, which was interpreted as an improvement of the gut microbiota composition. However, this study did not use a single bacterial strain, but the mixture of more than 50 microorganisms contained in kefir, and only reported a dose for total LAB (9.32 ± 0.23 log_10_ CFU/ml) and yeast (7.12 ± 0.36 log_10_ CFU/ml) (21). It is therefore difficult to calculate and compare the precise concentration of *Lk* that was administered in this study, and to infer the changes observed to a single potentially probiotic strain, as it could be attributed to several microorganisms and their potential synergism.

In addition, the composition of microorganisms in kefir may vary depending on its origin, the substrate used in the fermentation process, and the culture maintenance methods (41).

The product used in our study is a commercial preparation registered as a probiotic for human medicine, containing *Lk* with 30-day stability of reconstituted product guaranteed by the producer. Currently, there are no recommendations for the dosage of *Lk* in dogs. Daily dosages used in mice and people were 10^8^ and 10^10^ CFU, respectively (24, 26).

The dose administered to dogs in the present study was extrapolated from the dose for an adult person as recommended by the manufacturer. While the daily dose for people (five drops) should contain ≥10^9^ AFUs of live and viable *Lk*, the results presented here indicate that the same dose, administered to the dogs of this study, was more equal to 3.2 ± 2.4 × 10^7^ CFU.

The 30-day duration of the experiment was chosen based on available literature and a suspected washout time of 4 weeks after discontinuation of administration (42).

Fecal samples were chosen over other types of samples to evaluate the intestinal microbiota, as they can be collected in a noninvasive manner, raising no ethical concerns in comparison to, for example, intestinal mucosal biopsies. In addition, the ability of *Lk* to modulate microbiota composition has already been demonstrated using fecal samples in people and mice (25, 26). Similarly, concentrations of IgA from duodenal biopsies and fecal samples showed no difference in a previous study (4).

As for the gut microbiota, in line with what has already been reported for probiotic supplementations in healthy individuals (43), no changes were detected after *Lk* administration.

The IgA results showed a large interdog variability at T0 (from 1.33 to 35.35 mg/g of dehydrated feces), which, although all dogs appeared clinically healthy, could depend on the extreme variability in experimental dogs' signaling, age, life environment, and food taken.

Moreover, our IgA variability is in agreement with the data reported by other authors (29, 44).

Regardless of the basal value, we have noted the absence of a significant variation in the fecal IgA content comparing the experimental time points, i.e., pretreatment (T0), during (T15 and T30), and 30 days after *Lk* administration (T60).

Possible explanation of the results could be related to a poor immunomodulatory effect of *Lk* toward the canine GALT (gut associated lymphoid tissue) due to (a) poor *LK* viability in pharmaceuticals administered and consequent insufficient probiotic dose; (b) inadequate administration period; and (c) poor vitality in the gastrointestinal tract of the dog with lack of *Lk* probiotic activity in dogs.

Considering the first point, the differences found between the concentration declared by the company (*Lk* ≥ 25 × 10^9^ AFU/capsule; ≥ 10^9^ AFU/dose) and that found in our quality control analysis (81 ± 61 × 10^7^ CFU/capsule; 3.2 ± 2.4 × 10^7^ CFU/dose) must be emphasized.

It must be stressed that the gap between what was declared by the probiotic company and what was highlighted by an independent analysis is not a rare event. In fact, analyzing the literature, numerous papers, both in the field of human and veterinary medicine, evidence this gap, with several-folds reduction of live probiotics concentration with respect to which reported by the companies (45–47).

Though not much is known about the minimal dose and/or frequency of probiotics required for the probiotic effect, it seems to be dose-dependent (48). For this reason, it cannot be excluded that the absence of changes observed in fecal microbiota and IgA during the trial with *Lk* can be attributed to an insufficient dosage, corresponding to ~3% of the expected dose indicated for humans.

It is also true that an improvement in the enteric immune function in dogs was observed even after the administration of *Lactobacillus fermentum* at the dosage of 1 × 10^7^ CFU/daily dose for 1 week, similar dose, and lower trial time, than the one actually used by us (18).

With respect to the treatment time, our study treatment is longer than employed in similar studies (15–17), with two intratreatment withdrawals in addition to pre- and post-treatment sampling, so this point can be excluded as a cause of poor response too.

Lastly, no previous studies have analyzed the *Lk* vitality in the canine gastrointestinal tract; therefore, it cannot be excluded that this may be the cause of the poor probiotic activity observed in our study.

Conversely, studies performed in human medicine have verified the gastrointestinal *Lk* vitality showing a high rate of adhesion of *Lk* to intestinal cells and strong resistance to gastric juice and intestinal bile salts (49).

Limitations of the study include the fact that dogs did not receive a uniform diet during the experiment, and the relatively low number of dogs included, further confounded by the exclusion of dog #6 from the analysis.

However, we believe that not artificially standardizing the dogs' diet might allow for results to be translated easier into real-life veterinary practice conditions and would showcase that *Lk* is able to impact the microbiota independent of the diet given. We also believe that our methods of analysis will, to a certain degree, be able to counteract any dietary effect, as the dogs could serve as their own controls.

With respect to the sample size, the current study is similar to other feeding trials performing similar analyses (15–18).

In conclusion, our study was unable to demonstrate a significant change in microbiota composition or function in healthy dogs administered with *Lk* at a dose of 3.2 ± 2.4 × 10^7^ CFU/daily dose for 30 days. Further research will be necessary in order to assess the efficacy of a higher dose or of the combination of *Lk* with other potential probiotics.

## Data Availability Statement

The microbiota datasets generated for this study can be found in the Sequencing reads were deposited in the National Center for Biotechnology Information Sequence Read Archive (NCBI SRA; BioProject ID PRJNA 592436).

## Ethics Statement

The trial was authorized by the Animal Welfare Committee of the University of Bologna (Protocol No. 3885 of 21/07/2017). Written informed consent was obtained from the owners for the participation of their animals in this study.

## Author Contributions

AG and MP were responsible for the conception of the study and data interpretation and wrote the manuscript. AZ and MS performed fecal IgA determination. MF reviewed the manuscript and provided critical suggestions and comments. ST, MB, and PB analyzed fecal microbiota. RZ performed quality control analysis. All authors discussed the results and approved the final manuscript.

### Conflict of Interest

The authors declare that the research was conducted in the absence of any commercial or financial relationships that could be construed as a potential conflict of interest.
